# Silicon and Silver Low-Energy Ion Implantation into Titanium Plates for Improved Biocompatibility

**DOI:** 10.1021/acsomega.5c04495

**Published:** 2025-07-22

**Authors:** Estela K. Kerstner Baldin, Melissa Machado Rodrigues, Cristian Padilha Fontoura, Rafaele Frassini, Ana Elisa Dotta Maddalozzo, Jennifer Stefani Weber, Amanda Bohn, Klester dos Santos Souza, Célia de Fraga Malfatti, Mariana Roesch-Ely, Carlos Alejandro Figueroa, Cesar Aguzzoli

**Affiliations:** † Área do Conhecimento de Ciências Exatas e Engenharias, PPGMAT, 58802 Universidade de Caxias do Sul, Caxias do Sul, Rio Grande do Sul 95070-560, Brazil; ‡ Instituto de Biotecnologia, 58802 Universidade de Caxias do Sul, Caxias do Sul, Rio Grande do Sul 95070-560, Brazil; § Laboratório de Pesquisa em Corrosão (LAPEC), Universidade Federal do Rio Grande do Sul (UFRGS), Porto Alegre, Rio Grande do Sul 91501-970, Brazil; ∥ Instituto de Química, 28124 Universidade Federal do Rio Grande do Sul, Porto Alegre, Rio Grande do Sul 91501-970, Brazil

## Abstract

Surface modification of implant materials continues to address the issue of osseointegration. Moreover, combining osseointegration with bactericidal or antifouling properties in implants remains an open question for debate. Over the years, silver has been widely used as an agent for killing and preventing bacterial proliferation. Silicon, on the other hand, has been linked to improved osteogenic activity. In this work, titanium plates were incorporated with both Ag and Si ions through low-energy ion implantation, and surface characterization was carried out to validate the process. Ti plates containing 43 μg cm^–2^ Ag were further enriched with small amounts of Si, as verified by glow discharge optical emission spectroscopy (GD-OES) and energy-dispersive X-ray spectroscopy (EDS). This added step increased surface roughness by approximately 11% and led to a statistically significant difference in wettability, rendering hydrophilic features (from angles around 90° to below 70° for the Ag + Si condition)—both of which influence biocompatibility. Electrochemical tests showed a more reactive surface for implanted samples but nonetheless demonstrated stability over 28 days. Further research should focus on increasing the Si doping in Ti and evaluating subsequent *in vitro* conditions.

## Graphical abstract

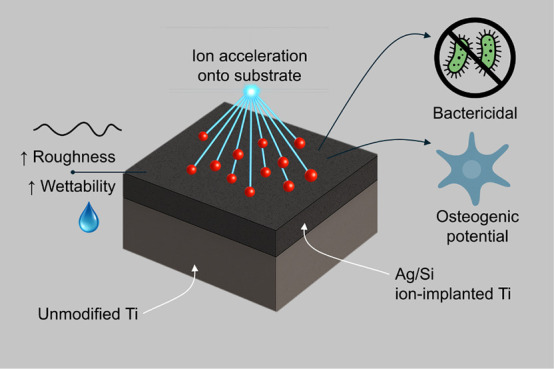

## 1 Introduction

In medicine, infection is a leading issue when it comes to the use of implants and other devices that come into contact with human tissue, accounting for 25.6% of all healthcare-related infections in the United States.^1^ In response , many efforts to eliminate the propagation of bacteria and other microorganisms have emerged in surface science, including the use of nanoparticles and coatings that inhibit biofilm formation and biofouling.^2^ Silver has been used as a bactericidal agent since antiquity and was vastly used to prevent infection and heal severe burns during the First World War, before the advent of antibiotics. Silver has established its role as one of the most promising and efficient doping or enriching metals for biomedical uses,^3^ but concerns arise as its use may damage mammalian cells.^4^

Enhancing surface properties also contributes to efforts to increase biocompatibility. In this sense, silicon has been cited as an osseointegration enhancer, usually allied with hydroxyapatite (HAP), as pointed out by some studies where osteogenic activity is improved,^5–8^ leading to bone growth in biological interfaces.^9^ Overall, Si has been recognized as a beneficial element in biomaterials.^10^

Ion implantation has become a popular method for doping or modifying the surface of biomaterials. Low-energy ion implantation (LEII) is identified as a preferable way for this surface processing, as the implanted dopant is set nearer to the surface compared to high-energy (beyond 40 and up to 500 keV^11,12^ ) ion implantation methods.^12^ This is particularly relevant for biological applications as surface interactions dominate. Furthermore, the small dosage is relevant to prevent the negative effects of Ag in humans, which is aimed at achieving antimicrobial properties. Additionally, these processes are eco-friendly as they generate little to no waste.

In this work, commercially pure titanium plates were incorporated with both silver and silicon ions via the Diversified Ion Plating (DIP) technique, which is based on low-energy ion implantation, and surface and biological characterization were carried out to evaluate the results. This work is based on previous research^13,14^ where Ag ions implanted showed significant bactericidal activity.

## 2 Materials and Methods

### 2.1 Sample Preparation

Samples were cut into square pieces of 20 × 20 mm and 0.3 mm in thickness from a commercially pure titanium sheet—grade 1, according to ASTM F67, provided by Sandinox and certified for use as a biomaterial.

Silver used for evaporation was in the form of pellets, had a purity of 99.99%, and was supplied by Kurt J. Lesker (USA). A wafer with an orientation of [100] was used for silicon evaporation.

Titanium plates were cleaned by immersion in pure acetone using an ultrasonic bath for 30 min. Afterward, the samples were air-dried and arranged in a sample holder for the low-energy ion implantation process.

### 2.2 Ion Plating

Low-energy ion implantation (LEII) was performed on DIP equipment. The process occurs in three different stages, divided as follows:

The Ag or Si pellets were placed into a graphite crucible and evaporated by means of an irradiated e-beam, which is generated by an electron gun placed beside it and directed into the crucible’s core through a magnetic field.
Evaporated material rises from the crucible and meets a second electron source, placed in the middle of the chamber, which collides with the vaporized atoms and ionizes them.
The substrates are placed above the second ion source in a sample holder that is subjected to a bias voltage (−2 or −4 kV for Ag and Si, respectively), which accelerates the resulting ions into the substrates. The ions are then implanted into the surfaces exposed to the beams. The exposure time for both conditions was 1 h. Samples implanted with Ag were named TiAg, while those implanted with both Ag and Si were named TiAgSi.

### 2.3 Monte Carlo Simulation

Monte Carlo simulation was performed to obtain the depth profile of Ag and Si ions in Ti by using the software Stopping and Range of Ion in Matter (SRIM 2013). The simulation parameters were simulated with the self-bias voltage for the acceleration of Ag and Si ion beams in the energies of 2 and 4 keV, respectively.

### 2.4 Surface Characterization

The topography of the substrate was also examined before and after ion implantation using a Tescan Mira 3 microscope and a field emission gun scanning electron microscope (FEG-SEM) at different zoom levels.

Microscale surface roughness was assessed through profilometry using a Model 112 Taylor Hobson profilometer. Nanoscale roughness was investigated by atomic force microscopy (AFM) in a SPM-9700 Shimadzu model. A pyramidal tip was used, composed of silicon nitride with a gold coating (TR800 Olympus), and it exhibits a resonant frequency range between 24 and 73 kHz and a spring constant in the range of 0.15–0.57 N m^–1^.

Contact angle was used to determine sample wettability and was obtained via the sessile drop technique with an SEO Phoenix 300 tensiometer. The resulting values are an average of at least 10 measurements carried out 1 day after implantation in different regions of samples. The fluids used for this assessment were distilled water and simulated body fluid (SBF), produced according to Kokubo’s protocol.^15^

### 2.5 Elemental Analysis

Rutherford backscattering (RBS) was used for quantitative elemental analysis. It was carried out in a Tandem ion accelerator, using a monoenergetic He^+^ 2 MeV ion beam and a detection angle of 165°. Experimental data were then compared with SIMNRA simulations of the sample and ion beam to investigate quantitative elemental analysis. Additionally, glow discharge optical emission spectroscopy (GD-OES) was used to investigate Si presence and implantation profiles in a GD-Profiler 2 (Horiba) at a pressure of 650 Pa and Ar^+^ bombardment with RF power of 15 W. Furthermore, the composition was simultaneously verified with SEM micrographs using a coupled silicon drift detector (Oxford Instruments X-act) for energy-dispersive X-ray spectroscopy (EDS) through composition maps in qualitative analysis.

For evaluating the migration of implanted ions into an SBF solution, inductively coupled plasma optical emission spectrometry (ICP-OES) was used with an ICAP 7000 series Thermo Scientific equipment for reading the method SMEWW 3120B, with a standard for curve plotting: Periodic Table Mix 1 for ICP (Sigma-Aldrich).

### 2.6 Electrochemical Measurements

Electrochemical studies were performed using potentiodynamic polarization and electrochemical impedance spectroscopy (EIS) techniques with Gamry Interface 1010E equipment. The samples were kept for 2 h until the open circuit potential (OCP) was reached before any electrochemical experiment. The electrochemical experiments were performed using a three-electrode setup with Ag/AgCl reference electrode, a platinum (Pt) wire as the counter electrode, and the samples (Ti, TiAg, TiAgSi) as the working electrodes. All potentials were referenced to the Ag/AgCl electrode.

### 2.7 Biological Properties

#### 2.7.1 Cytotoxicity Assay

Cytotoxicity was measured using the MTT (3-(4,5-dimethylthiazol-2-yl)-2,5-diphenyltetrazolium bromide) assay method that measures the integrity of the mitochondrial dehydrogenase enzyme through the formation of formazan crystals. Initially 5 × 10^4^ cells/mL (MG63) were seeded in 100 μL of DMEM culture medium supplemented with 10% fetal bovine serum (FBS) and 1% penicillin/streptomycin (P/S). Cells were incubated after 24 h in contact with the extraction solution obtained by immersing the samples for 1, 2, and 7 days. DMEM medium was used as the negative control; 1 mg·mL^–1^ of MTT was added to each well after removal of medium for 2 h. The formazan crystals were dissolved in 100 μL of DMSO (dimethyl sulfoxide) after removal of MTT solution. Reading was performed at 570 nm in a microplate reader (Spectramax M2e, Molecular Devices, USA), and the results were expressed as a percentage of viability, with the absorbance of the negative control set as 100% viability, and treated cells were calculated as a percentage of the control. Changes in cell viability were observed and recorded after 1, 2, and 7 days of exposure.

#### 2.7.2 Cell Adhesion and Spreading

MG63 cells were seeded in six-well plates at a density of 5 × 10^4^ cells·mL^–1^ on the samples for 1 and 2 days using 2000 μL of DMEM culture medium supplemented with 10% fetal bovine serum (FBS) and 1% penicillin/streptomycin (P/S). For fixation, cells were incubated with a 3% glutaraldehyde solution in PBS (v/v) for 15 min and dehydrated with 30, 50, 70, 90, and 100% (v/v) ethanol for 10 min at each concentration. Finally, the samples were kept in a desiccator until SEM/FEG and EDS analyses were performed.

#### 2.7.3 Cell Adhesion and Morphological Analysis

Cell adhesion and morphology were assessed using scanning electron microscopy (SEM) images acquired for each sample at 250× magnification (covering areas of approximately 1.2 mm^2^ field of view). Cell segmentation and counting were performed through an automated approach using a Python-based script. Images were converted to grayscale and enhanced for contrast to improve cell boundary visibility. Otsu’s thresholding was then applied to distinguish cells from the background, followed by contour detection to identify individual cells. For each detected cell, the area and perimeter were measured to compute the circularity index. Circularity analysis^16^ was conducted using the circularity index (*C*), which is defined as:

$$C=\frac{4\pi A}{P^{2}}$$

where *A* is the cell area and *P* is the cell perimeter. Values close to 1 indicate a perfect circle, whereas lower values indicate an elongated or irregular cell morphology. Objects with areas below a predefined threshold (10 square pixels) were excluded to minimize noise.

The total number of detected cells and their respective areas was recorded. The percentage of image area occupied by adhered cells (cell density) was calculated as the ratio of the total detected cell area to the total field of view (1.2 mm^2^), expressed as a percentage. Finally, visualization was performed by overlaying detected cell contours onto the original images, and circularity distributions were analyzed using histogram metrics.

## 3 Results and Discussion

### 3.1 Preliminary Analysis of Ion Implantation

The Monte Carlo simulations (Figure 1) provided preliminary insight into how the ion implantation of both Ag and Si takes place in Ti. The first observation is the gap between the depth and dose ion profiles of the two ionic species, as displayed by the estimated Gaussian profiles of incorporation into Ti. Ag shows a much narrower profile than Si, meaning that the ions are most likely packed together within 30 Å in Ti for a 2 keV implantation energy. A lower accelerating energy for Si would result in a greater mismatch between these profiles.

**Figure 1:**
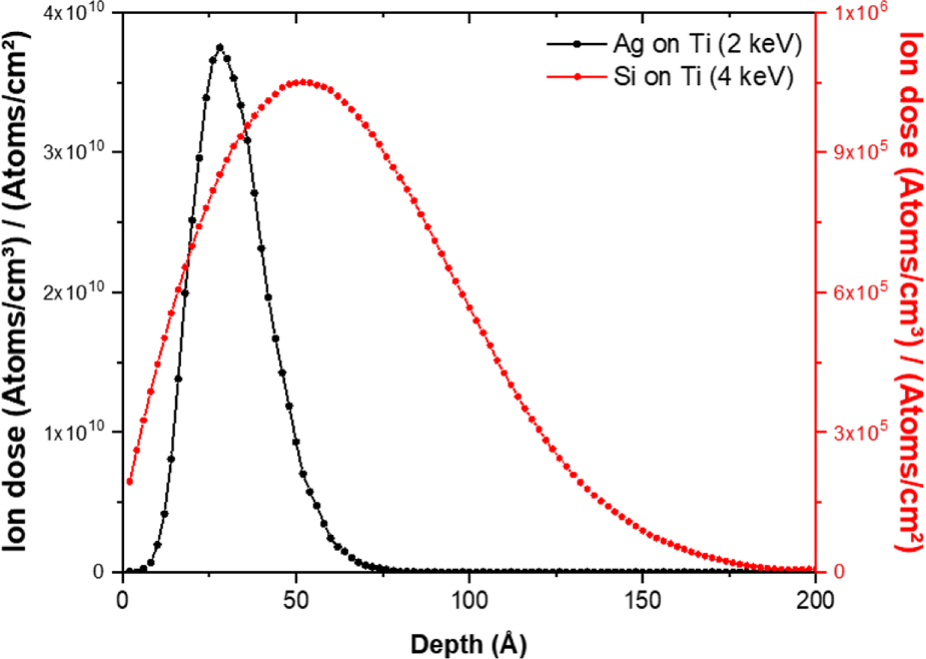
Monte Carlo simulations showing the in-depth silver and silicon concentration profiles inside titanium for implantation energies of 2 and 4 keV, respectively.

### 3.2 Elemental Analysis

RBS curves elucidate the chemical composition of the samples. Silver was detected at both 2 and 4 keV energies for ion implantation. For silicon detection, however, the technique does not provide enough resolution, as the ionic dose (seen in Figure 1—Monte Carlo simulation) is limited and the substrate masks its overall presence. The curves for both energies are displayed in Figure 2 for the sake of comparison.

**Figure 2:**
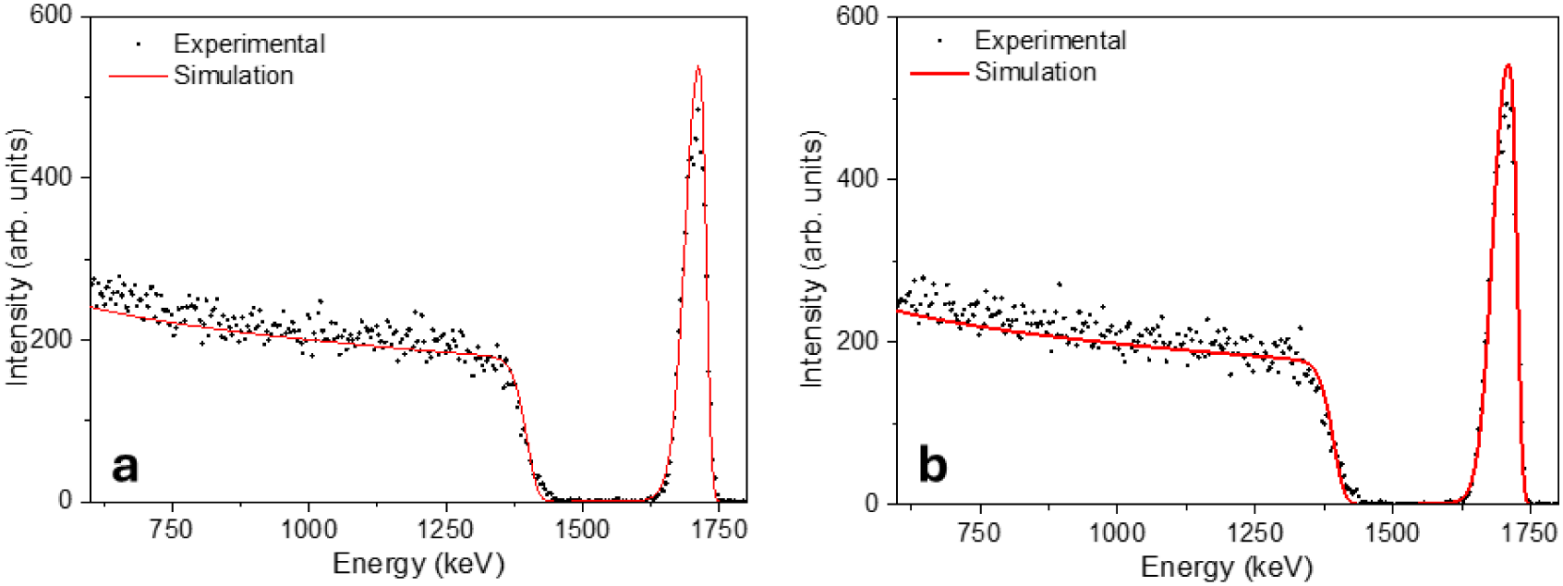
RBS spectra for 2 keV (a) and 4 keV (b) Ag ion implantation.

The mass for the two conditions did not vary significantly. For 2 and 4 keV, the areal density is, respectively, 43 and 48.4 μg cm^–2^ (both on the order of 10^15^ atoms·cm^–2^), indicating the low amount of silver obtained by the process. It is important to disclose that Ag may lead to several health issues, such as argyria, dermatitis, and eye irritation, besides having hepatic, renal, neurological, and hematological effects. The key factor lies in its dosage and tolerance, which is reported by the World Health Organization (WHO).^17^ This is further discussed in ICP-OES results, which examine how Ag is leached into a liquid medium.

For further silicon implantation, a 2 keV Ag implantation was chosen due to its lowest Ag content. As Si was not observed via RBS, GDOES and EDS analyses were also carried out. Figure 3a displays GD-OES profiles, which display two peaks near the surface, corresponding to Ag and Si within Ti. Monte Carlo simulations were used to estimate the depths of implantations and to calibrate the GD-OES profile depth. Additionally, EDS concentration maps display Ag and Si, for TiAg in Figure 3b and for TiAgSi in Figure 3c.

**Figure 3:**
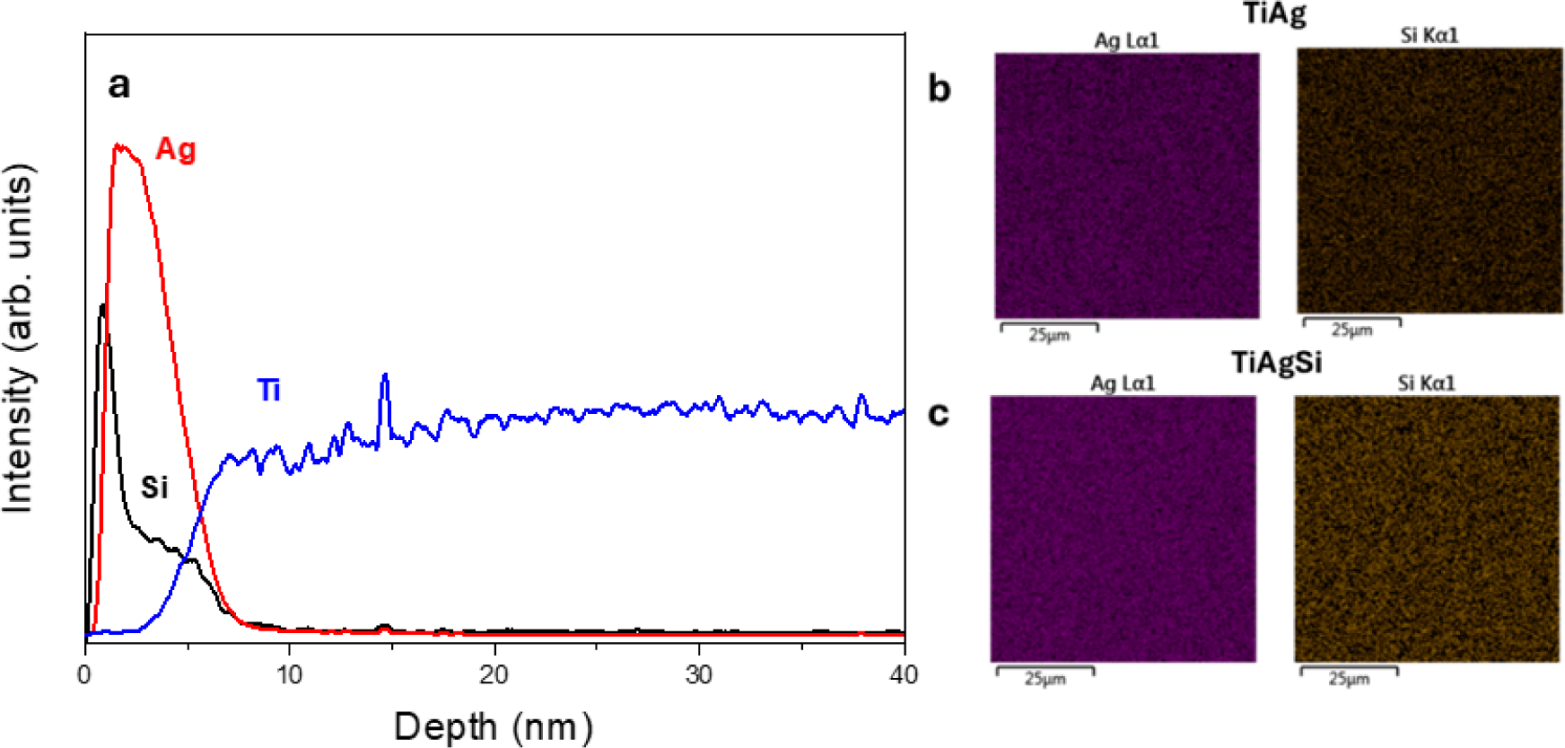
In-depth GDOES curves displaying two peaks near the surface corresponding to Si (4 keV) and Ag (2 keV) in TiAgSi (a) Ag and Si concentration maps for TiAg (b) and Ag and Si concentration maps for TiAgSi.

EDS was necessary for evaluating the presence of Si after ion implantation since RBS spectra could not capture the small amount of Si. Through concentration maps, it was observed that the presence of silicon was significantly increased for TiAgSi (Figure 3c), considering the residual Si observed for TiAg (Figure 3b). For the Si concentration map, it can be observed that it is uniformly distributed over the surface area analyzed by EDS, proving that the technique is successful in creating a homogeneous Si-doped surface.

### 3.3 Profilometry and Atomic Force Microscopy (AFM)

In terms of microscale roughness, for commercially pure titanium, an *R*_a_ roughness of 109 ± 13 nm and an *R*_z_ roughness of 761 ± 90 nm were measured. For samples containing both silver and silicon, there was an increase in roughness for both methods, with an average *R*_a_ of 122 ± 5 nm and an *R*_z_ of 848 ± 53 nm. The increase in roughness average is roughly 11%. This change was also noticed by AFM topography, where a statistically significant change was measured for TiAgSi (Figure 4).

**Figure 4:**
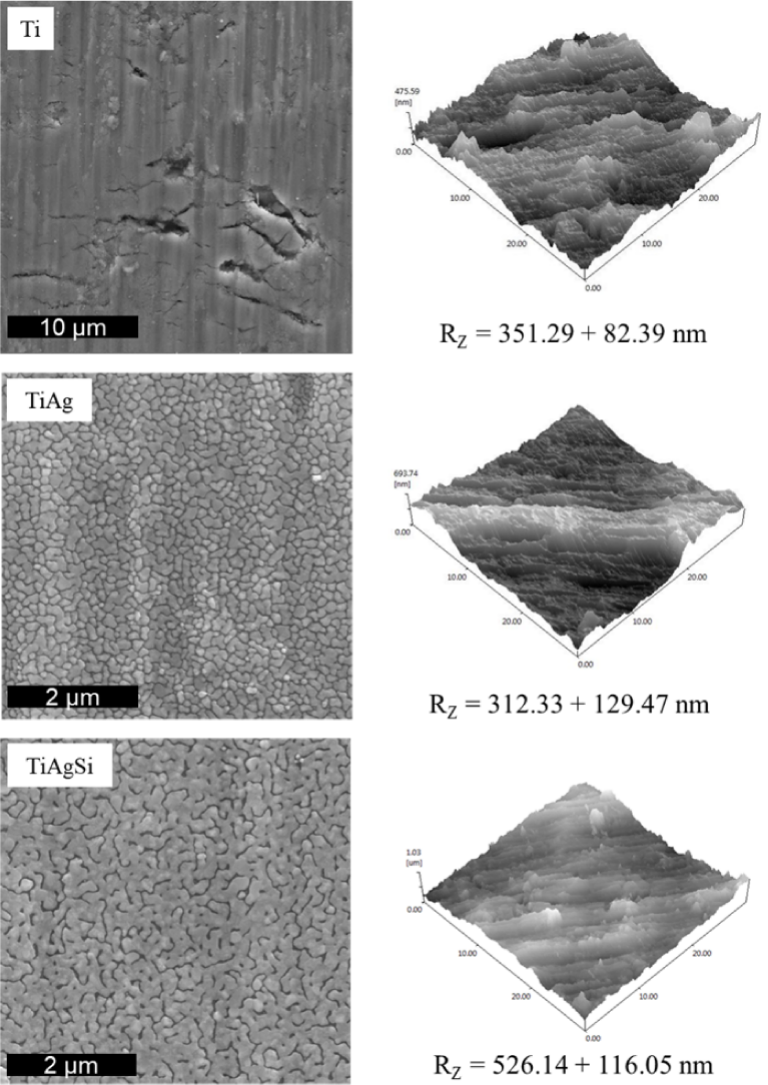
SEM micrographs for the different substrates and 3D AFM maps.

AFM readings provided an insight into nanoscale roughness for the modified substrates. The roughness of TiAg plates (*R*_z_ = 312.33 ± 129.47 nm) falls within the error range for pristine roughness (*R*_z_ = 351.29 ± 82.39 nm). Silicon incorporation, however, displayed a significant increase in roughness compared with the other two surfaces (*R*_z_ = 526.14 ± 116.05 nm). Increased surface roughness has often been correlated with enhanced osseointegration in biomaterials^18–20^ —a fact attributed to the larger surface area, which facilitates protein adsorption and, therefore, cell anchorage.^21^ This increase in roughness may occur because, when Si is implanted, the surface is already enriched with Ag, which can act as a diffusion barrier, preventing the deeper penetration of Si ions into the Ti substrate. This effect is evident in GD-OES depth profiles (Figure 3), where Si appears predominantly near the surface. Figure 5 schematically illustrates this condition, showing a shallower distribution of Si compared to Ag. Although Monte Carlo simulations (Figure 1) suggest that Si should penetrate deeper than Ag when implanted into pristine Ti, they do not account for the altered surface composition caused by prior Ag implantation. This discrepancy highlights the limitations of the simulation in replicating sequential implantation and supports the interpretation based on experimental data.

**Figure 5:**
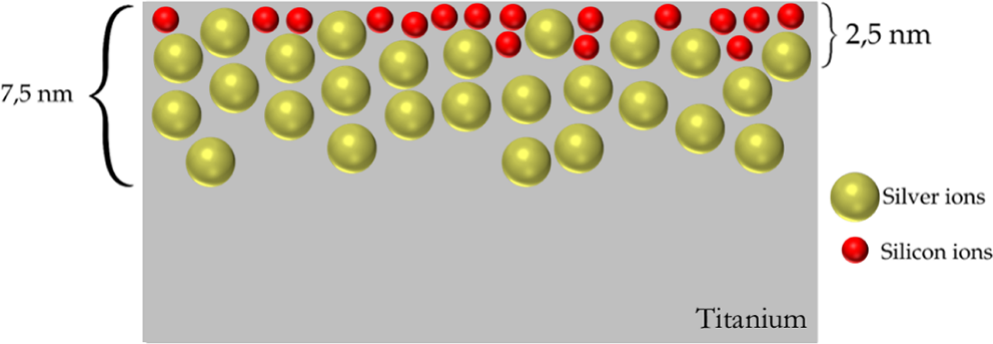
Schematic of Si and Ag implantation showing depth distribution.

Contact angle is an important feature to observe in biomaterials, as their interactions with water are predictive of how biological activity may occur—it is intrinsically related to surface roughness. For Ti, a contact angle near the hydrophobic range was detected, with an average of 89 ± 4°, and for TiAgSi a contact angle with mostly hydrophilic behavior was observed, with an average of 66 ± 6° (Figure 6).

**Figure 6:**
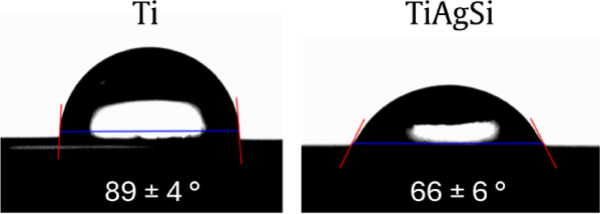
Contact angle measurements for Ti and TiAgSi samples.

This means that the Ti plates follow a Wenzel state,^22^ in which water completely wets the surface—following all patterns on its surface. Hydrophilicity is also linked to osseointegration and, therefore, is adequate for such purposes.

### 3.4 Ag and Si Ion Migration

Silver intake varies greatly from place to place. A normal level of silver in blood has been measured as <1 μg L^–1^ in individuals from the Melbourne metropolitan area. Daily intake has been reported as 0.4 μg day^–1^ in Italy, 7 μg day^–1^ in Canadian women, and 10–44 μg day^–1^ in the United Kingdom.^17^ The World Health Organization (WHO) explains that drinking water is a major source of oral exposure, as silver salts are used as bacteriostatic agents for treating water.^23^ Figure 7a shows the behavior of Ag leaching over periods of time ranging from 1 to 28 days. After exposure to the SBF solution, 17.25 ± 4.45 μg L^–1^ was obtained for a period of 24 h and for 28 days 68.15 ± 21.00 μg L^–1^. This suggests that the amount of leached Ag is well within the limit of 10 ppm (mg mL^–1^) that is reported for human cell toxicity.^24^

**Figure 7:**
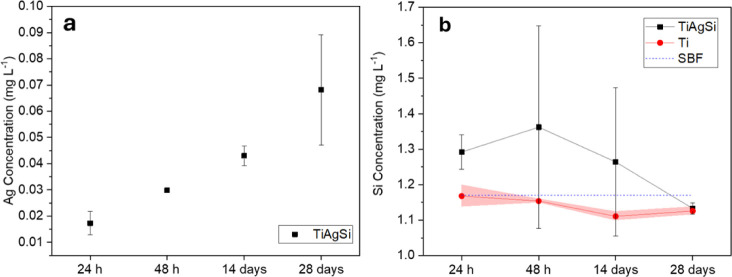
ICP-OES results showing Ag (a) and Si (b) migration into SBF over time.

In the case of prostheses or implants, further criteria should be analyzed, as sources vary in terms of Ag tolerance limits.^25^ As previously noted by Soares et al.,^13^ implanted Ag is in its metallic state, and its low ionization energy enables ionization even through contact with moisture or bodily fluids. This process facilitates its gradual disintegration, ultimately contributing to its antibacterial activity.

Silicon leaching from TiAgSi was compared to that observed in SBF fluid and the pristine Ti condition, as displayed in Figure 7b. A slight overall increase in leached silicon was observed for TiAgSi; however, variability among samples suggests that this difference is not statistically significant beyond the first day of contact with the solution. The shaded region represents the error for the pristine Ti condition.

### 3.5 Electrochemical Tests

Figure 8 shows open potential curves for 28 days in SBF. All of the samples presented a stable open-circuit potential (OCP) after 30 min of immersion and remained without significant changes during the 28 days of the experiment. The decrease in *E*_OCP_ with Ag and Si incorporation suggests a more active surface; however, the lower current density of TiAgSi compared with TiAg indicates that Si contributes to passivation, likely by forming a protective layer.

**Figure 8:**
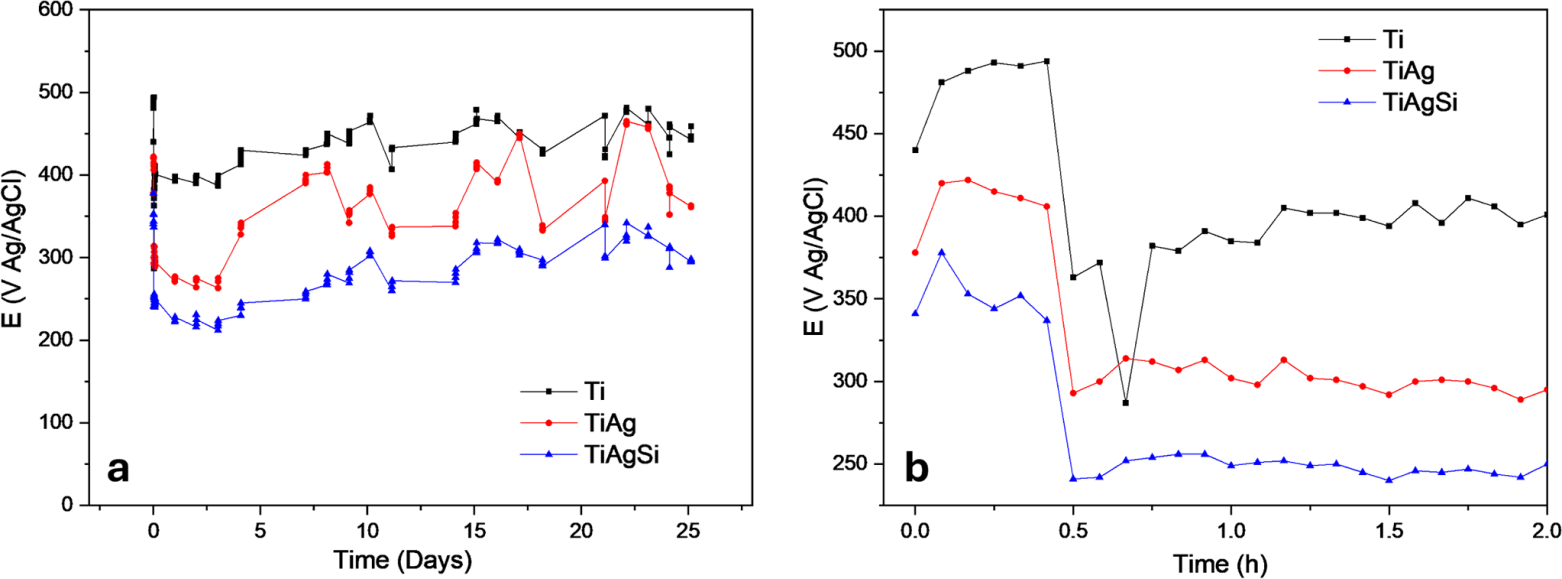
Open potential curves for Ti, TiAg, and TiAgSi over the period of 28 days (a) and 2 h (b).

The substrates presented similar corrosion potentials, with Ti presenting a slightly more positive potential and lower current density, which implies a more inert surface, consistent with the behavior of Ti. Tafel slopes (Figure 9) show that TiAg shifts toward a higher current density, indicating an increased corrosion rate. TiAgSi, despite exhibiting a more negative *E*_corr_, has a lower current density than TiAg, suggesting that Si incorporation enhances passivation and reduces the corrosion rate.

**Figure 9:**
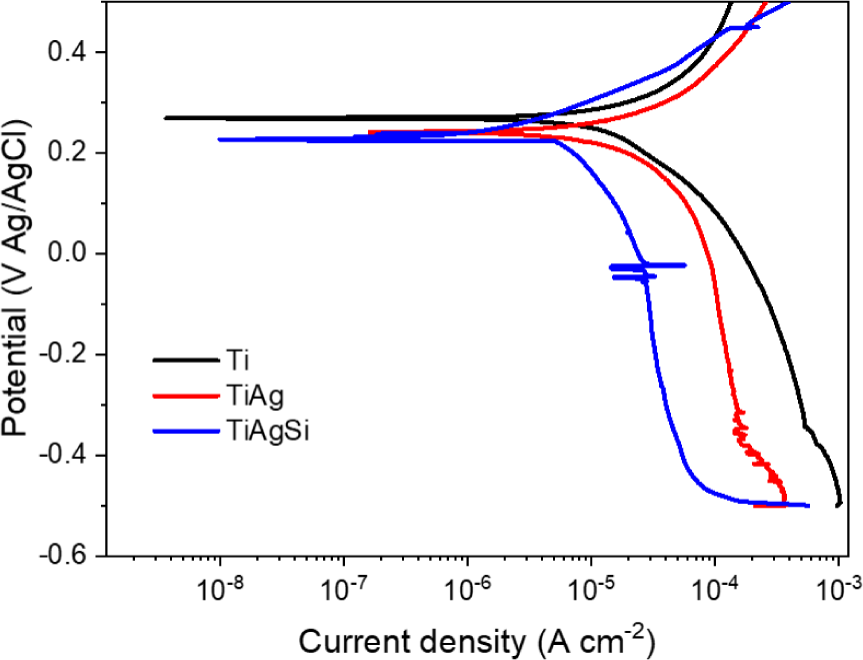
Tafel polarization curves of Ti, TiAg, and TiAgSi.

Overall, pristine Ti has better corrosion properties. TiAgSi has a lower current density than TiAg, which indicates that the Si incorporation is beneficial for providing a lower corrosion rate, most likely due to a passive layer.^26,27^ On the other hand, TiAgSi is less noble, as its potential is more negative than that of TiAg. This is probably due to Si solubility in Ti or TiO_2_, which may either weaken or improve corrosion properties, as it can be presented as a substitutional or interstitial solid solution.^26^

### 3.6 Biological Assays

#### 3.6.1 Cytotoxicity

The cytotoxic effect was assessed using the indirect MTT test, as shown in Figure 10. No reduction in cell viability was observed over periods of 1, 2, and 7 days in the presence of the extract from treated samples.

**Figure 10:**
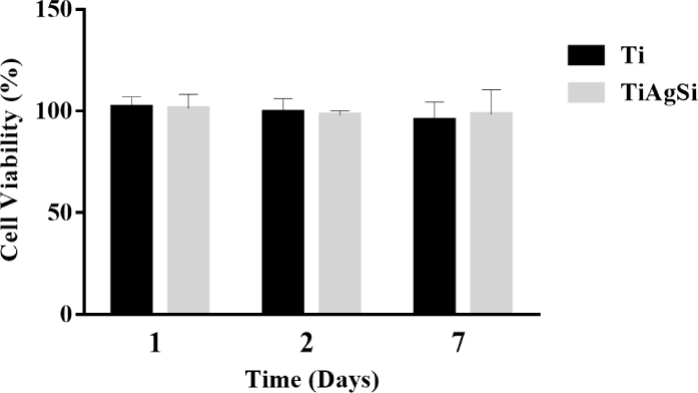
Cell viability results obtained through the indirect MTT test.

The threshold for toxicity is 70% of cell viability, following ISO standard 10993-5 (2009).^28^ All samples showed values above 70%, indicating that the samples did not exhibit toxicity to the extracts. This is consistent with previous cytotoxic assays conducted on Ti plates enriched with Ag.^13^

#### 3.6.2 Cellular Adhesion and Spreading

Cell adhesion was observed at two different times points: after 1 day and 2 days. These results are shown in Figure 11, which displays cell adhesion for pristine Ti and TiAgSi samples. The cells spread out well and maintained their spindle-like morphology, a behavior observed in Si-containing coatings over Ti.^29^

**Figure 11:**
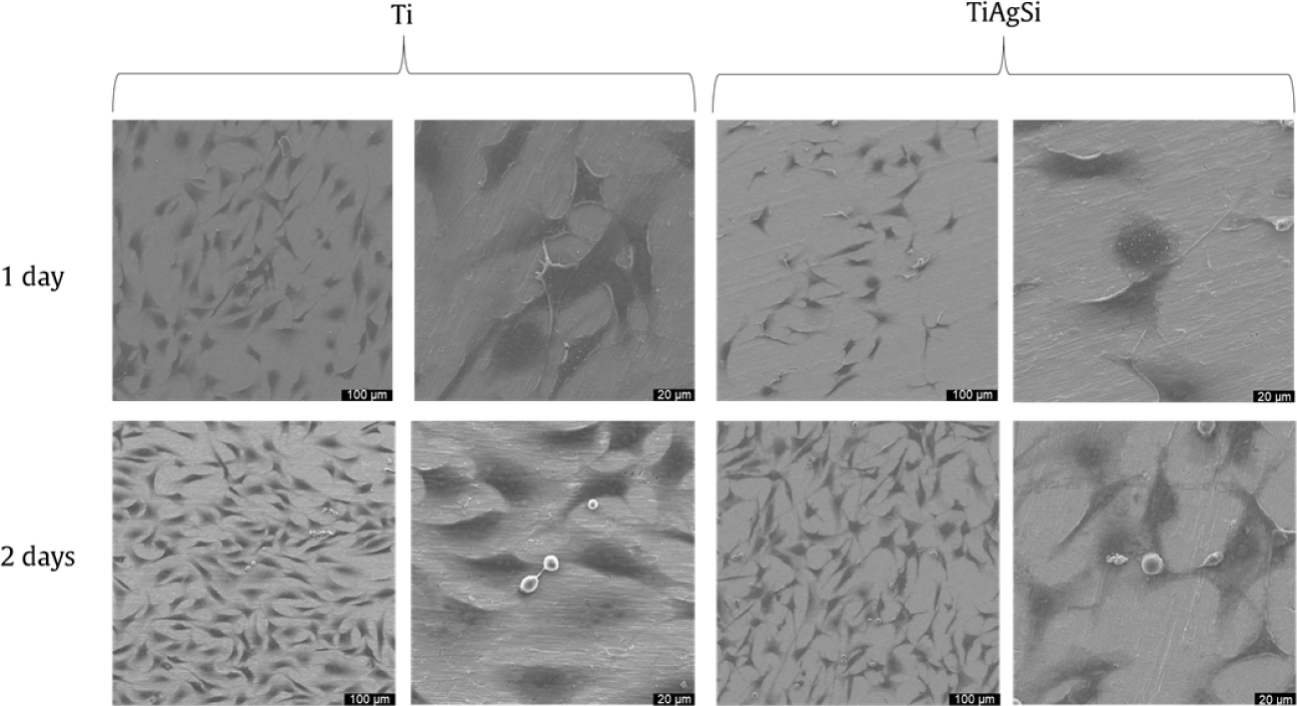
SEM analysis of samples in direct contact with MG63 cells.

Broader SEM micrographs were analyzed to quantify cell adhesion on both substrates (Figure 12), with cell contours highlighted, and the label number indicating the day. In regions of approximately 1.2 mm^2^, the cell count varied over time under both conditions. Pristine Ti exhibited higher initial adhesion (24 h) than TiAgSi. However, after 48 h, TiAgSi surpassed Ti in cell attachment, demonstrating an inverse trend between the two materials.

**Figure 12:**
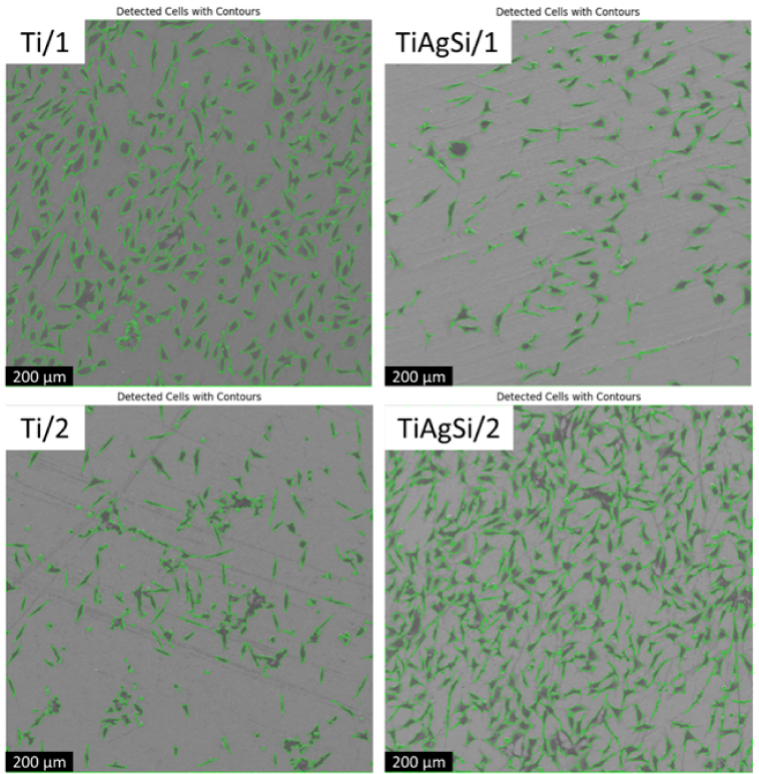
Cell analysis based on SEM images.

Table 1 summarizes key adhesion parameters, including cell count, area coverage, and circularity, all of which are critical indicators of cell–substrate interactions.

**Table 1:** Samples and Corresponding Cell Count, Area, and Circularity

Sample/Day	Cell count	Cell area (%)	Cell circularity (−)
Ti/1	329	20	0.19
Ti/2	217	6.9	0.27
TiAgSi/1	204	6.5	0.19
TiAgSi/2	315	24.0	0.22

While cell count on Ti decreased over time, TiAgSi exhibited the opposite behavior, with a significant increase after 48 h. This suggests that while Ti facilitates initial adhesion, it may not sustain long-term cell attachment, potentially due to weaker protein binding^30^ to extracellular matrix or lower surface stability. Conversely, TiAgSi appears to promote delayed but enhanced adhesion, possibly indicating improved proliferation or differentiation.

Additionally, cell circularity increased on Ti over time (0.19 → 0.27), whereas it remained more stable on TiAgSi (0.19 → 0.22). This suggests that cells on Ti may undergo rounding and detachment, whereas those on TiAgSi maintain a moderately spreading morphology, indicating stronger adhesion (Figure 12 and Table 1).

From this analysis, Ti is more effective for short-term adhesion, while TiAgSi exhibits superior long-term cell retention and spreading. The increased adhesion and area coverage on TiAgSi at 48 h suggest that its surface chemistry and topography modifications may enhance its suitability for long-term cell growth.^29^ This is especially true when we know that changes in topography—at nanoscale, for instance—will directly affect protein adsorption and, therefore, cell adhesion.^31^

Qualitative agar diffusion tests conducted under the same conditions containing only Ag ions have shown bacterial inhibitory activity against *E. coli* and a bacterial concentration reduction in industrial wastewater after exposure to the titanium plates, as Ag ions are leached to the medium.^13^ This suggests that the tailored TiAgSi may benefit from various surface characteristics that contribute to enhanced biocompatibility and antimicrobial properties of Ag.

## 4 Conclusion

Low-energy ion implantation is a technique that imparts desired properties to metallic substrates for use in biological contexts due to its low ion, as well as the ability to tune surface characteristics. The surface modification induces significant changes in many properties, such as roughness, wettability, corrosion resistance, and biological performance, as demonstrated in this study.

The use of Si as a doping agent for osseointegration has been debated over the years, and while it is a strong contender for enhancing osteogenic activity in such contexts, future studies should investigate higher dosages of Si—possibly through longer ion implantation durations at the same energies and conduct further biological testing. Additionally, new studies should explore other DIP process variations, such as using materials with osteogenic and antimicrobial properties.

Finally, the DIP technique is a clean and sustainable process that generates no hazardous residues and minimizes material waste. Its eco-friendly nature aligns with global sustainability goals, such as the United Nations Sustainable Development Goals and ESG principles, promoting responsible manufacturing and environmental stewardship. As industries seek greener alternatives, DIP offers an efficient and sustainable solution for advanced material processing.
